# Prevalence and associated factors of postpartum depression in Southwest, Ethiopia, 2017: a cross-sectional study

**DOI:** 10.1186/s13104-018-3730-x

**Published:** 2018-08-29

**Authors:** Sitotaw Kerie, Melak Menberu, Wondwossen Niguse

**Affiliations:** 1 Nursing Department, College of Medicine and Health Sciences, Bahir Dar University, Bahir Dar, Ethiopia; 2 Psychiatry Department, College of Medicine and Health Sciences, Bahir Dar University, Bahir Dar, Ethiopia; 3 Nursing Department, College of Medicine and Health Sciences, MizanTepi University, Mizan Teferi, Ethiopia

**Keywords:** Postpartum depression, Postpartum, Associated factors, Ethiopia

## Abstract

**Objective:**

The aim of this study was to determine the prevalence and associated factors of postpartum depression among mothers who gave birth within the last 12 months among hospitals of Southwest Ethiopia, 2017.

**Result:**

The study revealed that 138 (33.82%) of mothers had postpartum depression. Unplanned pregnancy adjusted odds ratio (AOR) = 4.49, 95% CI (2.31, 8.71), age from 15 to 24 years AOR = 0.420, 95% CI (0.18, 0.98), having a chronic physical illness AOR = 7.71, 95% CI (2.34, 25.44), experiencing death of infant AOR = 4.12, (1.78, 9.51) and unstable marital condition AOR = 6.02, (2.79, 12.99) were significantly associated with postpartum depression. The prevalence of post-partum depression was found to be high. Therefore urgent attention must be given to this problem, in particular towards its early detection, so that morbidity could be reduced in this group of women.

## Introduction

Postpartum depression is a mood disorder with symptoms include changes in sleep and eating patterns, fatigue, sadness, crying, anxiety and guilty feeling related to ability to care the infant [1]. According to standardized diagnostic and statistical manual, postpartum depression is one type of depressive episode, which occur within 1 year of childbirth. It is a significant public health problem which affects about 17% and 19% mothers globally and in low and middle income countries respectively [2, 3]. Regarding the associated factors anxiety during pregnancy, stressful recent life events, poor social support, previous history of depression, early life abuse, abuse by an intimate partner, maternal low educational attainment, low socioeconomic status at the time of pregnancy, and a history of mental illness have been associated with postpartum depression [3, 4].

Untreated postpartum depression has serious adverse long term effect on both mothers and their children’s. For the mother, the episode can be the precursor of chronic recurrent depression. On the other hand, for her children, it can contribute to emotional, behavioral, and cognitive problems in later life [5, 6]. Since depressed mothers stop breastfeeding earlier, the infants are more likely to have episodes of diarrhea, poor mother–infant relationship, and can affect child development and other infectious illness [1, 7].

Although 90% of the world’s children live in low and middle-income countries, little is known about the prevalence rate of postpartum depression in Ethiopia. Mental and neurological conditions like postpartum depression contribute more than 12.3% disability adjusted life years [8]. Thus, reliable estimates of postpartum depression in these contexts are required for the development of national and international health policies [2]. Therefore, the purpose of this study was to determine the prevalence and associated factors of postpartum depression to fill the gap.

## Main text

### Methods

#### Study setting, design, period and participants

An institutional based cross-sectional study was conducted at Mizan-Tepi University teaching hospital, Gebretsadik Shawo general hospital and Tepi primary hospital in Southwest Ethiopia from 1st December to 1st February 2017. This site is located 561 km from Addis Ababa. Monthly, Mizan-Tepi University teaching hospital, Tepi primary hospital and Gebretsadik Shawo general hospital provide postpartum service for 256, 234, and 245 mothers respectively. All delivered mothers within the last 12 months and those mothers who have postpartum follow up in these three hospitals were source population. All delivered mothers who have postpartum follow up during the study period in these hospitals were included in the study.

#### Sample size determination and sampling procedure

The sample size was calculated using single population proportion formula [9], by considering the following assumptions; proportion of postpartum depression (P) = 50%, the level of confidence (CL) = 95%, margin of error (d) = 5% and 10% non-response rate. Finaly by considering 10% non-response rate, the sample size was 422.

**Fig. 1:**
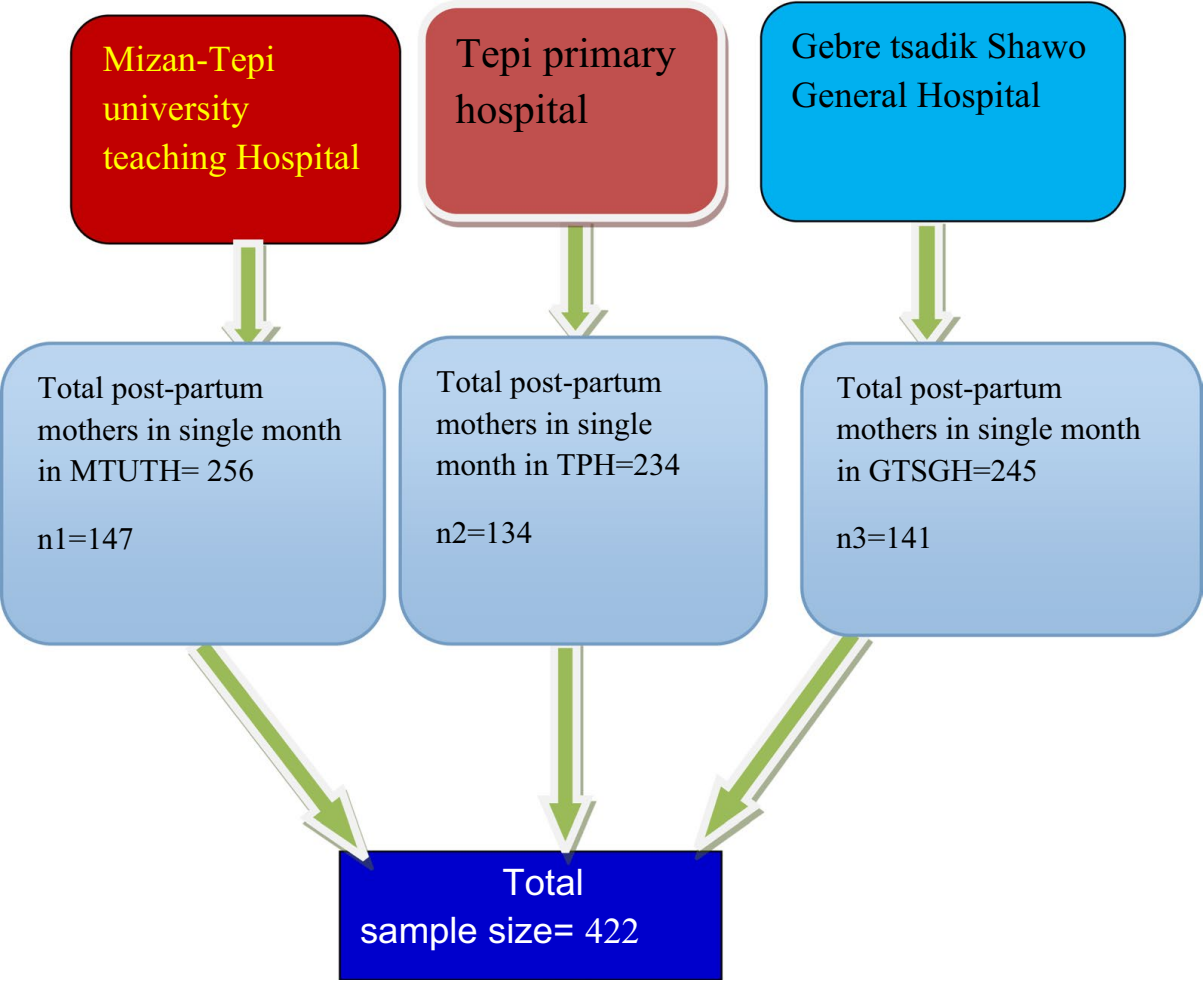
Sampling technique for prevalence and associated factors of post-partum depression among Mizan-Tepi university teaching hospital, Tepi primary hospital and Gebretsadik Shawo general hospital Southwest, Ethiopia, 2017

Mizan-Tepi University teaching hospital, Gebretsadik Shawo general hospital and Tepi primary hospital was selected as a study site primarily. The sample size was proportionally allocated to these three hospitals. Systematic sampling technique was used to select participants by considering the Kth value. The Kth value was calculated by dividing the total number of postpartum mothers to sample size, which was found to be two. Then the 1st comer mother was considered as first participant. Finally, the other participants were interviewed in every other interval until the required sample size is fulfilled (Fig. 1).

#### Measurement

The outcome variable (postpartum depression) was assessed by using an Edinburgh postnatal depression scale. The cutoff points to assess postpartum depression in this specific population was scored ≥ 10. EPDS has 10 items and each item has 4 Likert scales and it has a maximum score of 30 and minimum scores of zero. EPDS has a sensitivity of 80% and specificity of 84.4% [10]. The stress full life event was assessed by using perceived stress scale. Perceived stress scale has minimum of 0 and maximum of 40 scores and has 5 Likert scales with the Coefficient alpha reliability of 0.85. Scores ranging from 0 to 13 would be considered low stress, scores ranging from 14 to 26 would be considered intermediate stress and scores ranging from 27 to 40 would be considered high stress [11]. Social support was assessed by using the 3-Item Oslo social support scale and scores 3–8 = poor social support, 9–11 = intermediate social support and 12–14 = strong social support with Cronbach’s alpha of 0.60 [12].

#### Statistical analysis

Data was entered by using Epi Info Data Version 3.5.1 and exported to SPSS 21 statistical package for analysis. The first association between each independent variable and dependent variable was assessed in bivariate analyses. Then, those independent variables with *P* value < 0.25 were transported to multivariate logistic regression to control the cofounders and to identify predictors of postpartum depression. A P-value of < 0.05 was used as the criterion for statistical significance and OR with 95% confidence interval was used to indicate the strength of association [9].

### Results

#### Socio demographic data of respondents

Of the total 422 eligible mothers, 408 were participating in the study, which made a response rate of 96.7%. Among the respondents, 183 (44.9%) were in the age range of 15-24 years with mean age of 26.06 years with a standard deviation of ± 5.79. Thirty seven percent were Kefa by ethnicity. Majority 323 (79.2%) was currently married. Regarding to the educational status, 156 (38.2%) respondents were primary level educated. Around 44% were orthodox religion followers (Table 1).

**Table 1:** Socio-demographic data of study population in Mizan-Tepi University Teaching Hospital, Tepi primary hospital and Gebretsadik shawo general hospital, 2017 (n = 408)

Variable	Frequency	Percent (%)	PPD	
			Yes	No
Age in years				
15–24	183	44.9	61 (33.3%)	122 (66.7%)
25–34	174	42.6	44 (25.3%)	130 (74.7%)
≥ 35	51	12.5	33 (64.7%)	18 (35.3%)
Religion				
Orthodox	178	43.6	47 (26.4%)	131 (73.6%)
Protestant	146	35.8	57 (39%)	89 (61%)
Muslim	82	20.1	34 (41.5%)	48 (58.5%)
Others	2	0.5		2 (100%)
Marital status				
Married	323	79.2	68 (21.1%)	255 (78.9%)
Single	57	14	45 (78.9%)	12 (21.1%)
Divorced	23	5.6	21 (91.3%)	2 (8.7%)
Widowed	5	1.2	4 (80%)	1 (20%)
Educational status				
Who cannot read and write	86	21.1	27 (31.4%)	59 (68.6%)
Who can read and write	65	15.9	36 (55.4%)	29 (44.6%)
1–8 grade	156	38.2	55 (35.3%)	101 (64.7%)
9–12 grade	62	15.2	10 (16.1%)	52 (83.9%)
Diploma	32	7.8	4 (12.5%)	28 (87.5%)
Degree and above degree	7	1.7	6 (85.7%)	1 (14.3%)
Ethnicity				
Kefa	151	37	30 (19.9%)	121 (80.1%)
Bench	114	27.9	44 (38.6%)	70 (61.4%)
Sheka	64	15.7	15 (23.4%)	49 (76.6%)
Amhara	36	8.8	24 (66.7%)	12 (33.3%)
Oromo	26	6.4	22 (84.6%)	4 (15.4%)
Tigre	17	4.2	3 (17.6%)	14 (82.4%)
Job				
Farmer	218	53.4	41 (18.8%)	177 (81.2%)
Private	66	16.2	24 (36.4%)	42 (63.6%)
Jobless	48	11.6	29 (60.4%)	19 (39.6%)
Daily worker	43	10.5	35 (81.4%)	8 (18.6%)
Gov’t worker	31	7.6	9 (29%)	22 (71%)

*PPD* post-partum depression, *gov’t worker* government worker

#### psycho-social related factors of respondents

Among respondents, 24.8% of them had currently unstable marital condition. And, nearly thirty percent of them had unplanned pregnancy, 127 (31.1%) of them had unwanted pregnancy, more than half of them 210 (51.5%) had a life with an intermediate stress level and one quarter of them did not get any social support during their postnatal period. In other hand, husbands of 88 (21.6%) mothers had additional sexual partner.

#### Clinical factors of respondent

Among 408 respondents 87 (21.3%) had previous history of depression, 30 (7.4%) had chronic medical illness, 77 (18.9%) of were giving birth to low birth weight, 100 (24.5%) of them were facing difficulty of breast feeding, 111 (27.2%) were not given spontaneous vaginal delivery and among all mothers 17.2% of them experienced the death of infant.

#### Prevalence of postpartum depression

Among 408 respondents 138 (33.82%) of them full fill criteria for post-natal depression.

#### Factors associated with postpartum depression

In multivariate logistic regressions; those who have an unplanned pregnancy were 4.49 times more likely to develop post-partum depression as compared to planned pregnancy (AOR = 4.49, 95% CI 2.31, 8.71), those whose age is from 15 to 24 years are 58% times less likely to develop post-partum depression than those who has age greater than 30 years (AOR = 0.420, 95% CI 0.18, 0.98), Participants who have other chronic physical illness were 7.71 times more likely to develop post-partum depression than those who were not chronically ill (AOR = 7.71, 95% CI 2.34, 25.44), Participants who have been experiencing death of infant were 4.12 times more likely to develop post-partum depression than those who have no experiencing death of infant (AOR = 4. 12, 1.78, 9.51) and unstable marital condition (AOR = 6.02, 2.79, 12.99) were significantly associated with postpartum depression (Table 2).

**Table 2:** Logistic regression of associated factors and postpartum depression in Mizan-Tepi University Teaching Hospital, Tepi primary Hospital and Gebretsadikshawo General Hospital, 2017 (n = 408)

Variable	Postpartum depression		COR (95% CI)	AOR (95% CI)	P-values
	No (n)	Yes (n)			
Age					
15–24	122	61	0.27 (0.14, 0.52)	*0.420* (0.18, 0.98)	0.001
25–34	130	44	0.19 (0.15, 0.36)	*0.19* (0.19, 0.46)	0.002
≥ 35	18	33	1	1	
Unplanned pregnancy					
Yes	33	89	13.05 (7.88, 21.66)	*4.49* (2.31, 8.71)	0.001
No	237	49	1	1	
Chronic illness					
Yes	6	24	9.26 (3.69, 23.27)	*7.71* (2.34, 25.44)	0.002
No	264	114	1	1	
History of depression					
Yes	23	64	9.29 (5.4, 15.98)	0.95 (0.39, 2.32)	0.73
No	247	74	1	1	
Mode of delivery					
No	80	31	0.69 (0.43, 1.11)	0.65 (0.34, 1.25)	0.18
Yes	190	107	1	1	
Death of infant					
Yes	13	57	13.91 (7.25, 26.71)	*4.12* (1.79, 9.51)	0.003
No	257	81	1	1	
Current marital problem					
No	245	63	0.09 (0.05, 0.15)	*6.02* (2.79, 12.99)	0.001
Yes	25	75			

Significantly associated variables are in italics

### Discussion

This facility based cross-sectional study with the objective of the assessment of postnatal depression and its associated factors in the selected public hospitals, Southern Ethiopia was assessed the level of postnatal depression and the exposure variables impacting postnatal depression.

This study demonstrated that about 33.82% of the participants had postpartum depression. The finding of this study is comparable with studies done in Nepal which is 30% [13]. Perhaps this might be due to methodological similarity and use of the same tool with a similar cutoff point which is EDPS ≥ 10. Furthermore, this study is inline with a study conducted in Pakistan, which was 33.1% [14]. This might be due to use of similar study design, though the cutoff point used in Pakistan is different that was EDPS ≥ 12.

On the other hand, the result of this study is higher as compared to studies conducted in India 22% [15], in Czech Republic 10.1% [16]. This is might be due to the difference in study design and the time of evaluation. The study conducted in India was a systematic review and meta-analysis, whereas our study design was cross-sectional. Additionally, there is a variation in the time of the evaluation the study in India included studies published from the year 2000 up to 2016. In the last 16 years, many things have changed in Ethiopia, like economical inflation. The inflation can cause postpartum depression by making the living style stressful.

A study conducted in the Czech Republic was longitudinal and included the positive answer to question number eight, which refers to mood problems to determine the presence of depressive symptoms.

The finding of the current study also higher than the studies conducted in Iran 6.9% [17], in University of Oulu central Finland 22.2% [18]. This great variation might be because of the difference in study design, use of different cutoff point of EDPS score. The study conducted in Iran was a longitudinal cohort study and the cutoff point of EDPS score they used was ≥ 12. A study conducted at the University of Oulu central Finland was also a prospective longitudinal follow-up and used the cutoff points of EDPS score of ≥ 12 and included the Beck Depression Inventory (BDI) and General Health Questionnaire (GHQ) in addition to EDPS. The use of different cutoff points of the EPDS can give a difference prevalence of postpartum depression. But this finding was lower than from studies done in Uganda 43% [19], Asia, 63.3% [20], South India 45.5% [21]. The difference might be due to methodological variations between studies and differences in sociocultural, economical, health and health service utilization characteristics between respondents of the referenced areas and the study place.

In this study an unplanned pregnancy was significantly associated with post-partum depression. Participants who have an unplanned pregnancy were 4.49 times more likely to develop post-partum depression as compared to planned pregnancy. This is congruent with the study conducted in US [22], Isfahan city [23], northeastern Brazil [24], Pennsylvania [25], Czech Republic [16]. This might be due to unplanned pregnancy have a greater effect on maternal health by negatively affect mothers psychology and it can also bring economical burden and social judgement, those inturns can bring postpartum depression.

Age of participants was also significantly associated with post-partum depression. Participants whose age range are from 15 to 24 years are 58% times less likely to develop post-partum depression than those who has age greater than 30 years. Which is similar to finding in the study conducted in Nepal [13] and with other studies conducted in Joensuu, a town in Eastern Finland [26], China [27], US [22], university of Oulu, central Finland [18]. This decrement of postpartum depression in young age in this study can be due to increment of educational coverage currently in Ethiopia especially in young age which inturn increase ANC services utilization. This attributed to the fact that ANC service utilization gives promotion of health, prevention and treatment of diseases. Specifically birth preparedness and complication readiness is increased directly with ANC coverage, and as a result of this postnatal depression can be reduced in this age group. The other reason might be a great focus for maternal health has been given for the last 27 years and this can prevent other illness that causes postpartum depression. Chronic illness was another variable which was significantly associated with postpartum depression. Participants who have other chronic physical illness were 7.71 times more likely to develop post-partum depression than those who were not chronically ill. This finding is inline with similar studies conducted in Beirut [28], US [29], Joensuu, a town in Eastern Finland [26], China [27]. Chronic illness makes them economically dependant, brings public stigma due to life long treatment, an incurability of the disease can make them hopelessness, and getting birth during illness also another burden. Another reason might be; if there is chronic disease, appetite become decreased and they lack amino acids which are the precursor for serotonin. A lack of serotonin is one biological cause for depression.

Other variables which were significantly associated with post-partum depression were the death of infant and marital problems. This is consistent with studies done in India [30], Bruit [28], Uganda [31], Bangladesh [32], Spain [33], Nablus, Palestine [34]. One of the main cause for depression is loss of significance person in the life. Infant has a great significance in mothers’ life. Because of these mothers who lost their infant to become depressed during their postpartum period. The unstable marital condition can increase emotional distress and emotional distress facilitates the development of postpartum depression.

### Conclusion

The prevalence of post-partum depression in Southwest Ethiopia was found to be high. Age of the participants, unplanned pregnancy, chronic illness, death of infant and current marital problem were predictors of postpartum depression. Therefore urgent attention must be given to this problem, in particular towards its early detection, so that morbidity could be reduced in this group of women.

## Limitation

This research might be subjected to certain limitations. Data were not collected regarding different substance abuse, pharmacological lifetime treatment for depression, the recent history of antidepressants use or data of bipolarity. Study participants might not remember and report information correctly. The other limitation of this study might be the use of EPDS tool, use only for screening of depressive symptoms without a clinical diagnosis and use of the 3-Item Oslo social.

## Abbreviations

ANC: antnatal care
BDI: Beck Depression Inventory
EPDS: Edinburgh postnatal depression scale
GHQ: General Health Questionnaire
IRB: Institutional Review Board
PSS: percived stress scale
US: United States
